# NIA Division of Extramural Activities

**DOI:** 10.1093/geroni/igab046.1045

**Published:** 2021-12-17

**Authors:** Kenneth Santora

**Affiliations:** National Institute on Aging, Bethesda, Maryland, United States

## Abstract

Dr. Santora will discuss the work of the NIA Division of Extramural Activities. Dr. Santora will also be available for small group discussion.

